# Safety and Effectiveness of Blind Percutaneous Liver Biopsy: Analysis of 1412 Procedures

**DOI:** 10.5812/kowsar.1735143x.4388

**Published:** 2012-01-20

**Authors:** Aleksandra Szymczak, Krzysztof Simon, Malgorzata Inglot, Andrzej Gladysz

**Affiliations:** 1 Department of Infectious Diseases, Wroclaw Medical University, Wroclaw, Poland

**Keywords:** Liver Biopsy, Biopsy Complications, Liver, Chronic Diseases, Clinical Skills

## Abstract

**Background:**

Percutaneous liver biopsy is one of the most important and widely used methods for diagnosing chronic liver diseases; however, controversies related to the potential risk of complications and patient discomfort still exist.

**Objectives:**

The objective of this study was to evaluate the safety and success rate of blind percutaneous liver biopsy.

**Patients and Methods:**

We conducted a retrospective analysis of 1412 blind percutaneous thick-needle liver biopsies performed during 1977-2000 at a single center on 1110 patients, using archived medical data of the center.

**Results:**

The overall success rate of obtaining a liver sample with this method was 95.3%. Of all the samples assessed, 91.7% were determined to be fully representative for an evaluation by the pathologist. Complications occurred in 259 procedures (18.3%). While no fatalities associated with liver biopsy were noted, 9 serious complications (0.64%) directly related to biopsies were reported. Pain was the most common complication (15.3%). Significantly more complications (pain and vasovagal reactions) were reported in females (22.1%) than in males (16.1%) (P = 0.005). The rate of complications was significantly correlated with the stage of fibrosis (P = 0.027), i.e. the higher the fibrosis stage, the higher the complication rate. Previous surgical procedures involving the abdominal cavity or thorax influenced the effectiveness of liver biopsy (P = 0.017). Less operator experience was significantly associated with a higher rate of procedure failure (P = 0.002). Statistical significance of the relationship between individual operator efficiency and complication rate (P = 0.000) and that between individual operator efficiency and biopsy failure rate (P = 0.002) was observed.

**Conclusions:**

Blind percutaneous liver biopsy is a safe and effective invasive procedure, despite the fact that noninvasive fibrosis assessment methods are currently widely available and used instead of histological evaluation. Complications risk and failure rate are low if indications and contraindications are considered carefully and the biopsy is performed by a skilled and experienced operator. Certain groups of patients may benefit from an image-guided procedure to improve its effectiveness.

## 1. Background

Percutaneous liver biopsy is one of the most important and widely used methods for diagnosing chronic liver diseases. Indications for liver biopsy have evolved with the development of new molecular and serologic tests for the detection of liver diseases. In recent years, noninvasive techniques for the assessment of fibrosis have been developed and widely used. However, only histological examination provides detailed information regarding elements of liver histopathology, including identification of particular types of inflammatory cells in infiltrates, storage of pathological substances (i.e. iron, copper), lesions in transplanted organs. Similar to other invasive procedures, a potential risk of complications, including death, is associated with liver biopsy. The mortality rate, mainly because of hemorrhagic complications, varies from 0% to 0.33% depending on the cohort analyzed [1][2][3][4][5]. Bleeding in the peritoneal cavity and biliary peritonitis following puncture of the gall bladder or large bile duct are the most serious complications. The most common complications include pain of varying severity and vasovagal reactions. Intrahepatic or subscapular hematomas are also observed in some cases. Other rare complications include pneumothorax, bleeding in the pleural cavity, puncture of adjacent abdominal organs, infection, and breaking of the biopsy needle [2][6]. Accuracy of histological evaluation is directly dependent on adequate tissue samples [7][8]. Currently, image-guided biopsies are widely used to maximize the effectiveness of the procedure, although their superiority over blind biopsies in practice is controversial [9][10][11][12]. Despite long-term clinical experience and established indications and contraindications, the role of liver biopsy in the diagnosis and management of chronic liver diseases remains debatable. Controversies mainly involve the potential risk of complications and discomfort to patients, particularly in terms of the availability of contemporary noninvasive diagnostic techniques.

## 2. Objectives

An evaluation of the safety and success rate of blind percutaneous liver biopsy based on retrospective single-center data is presented in this paper.

## 3. Patients and Methods

We performed a retrospective analysis of archived medical data of patients with chronic liver diseases, on whom percutaneous thick-needle liver biopsy was performed as part of a standard diagnostic algorithm. All patients were hospitalized in the Department of Infectious Diseases, Liver Diseases and Acquired Immunodeficiencies of Wroclaw Medical University from 1977 to 2000. Data collected from archived medical documentation included age, sex, indication for liver biopsy, laboratory test results, complications and outcomes, success of procedures, results of histological evaluation of tissue samples, and name of the operator and his/her experience in performing biopsies. Assessment of procedure success and obtaining a representative sample was based on the opinion of the pathologist. All patients gave the informed consent for liver biopsy, recorded in medical documentation. As preparation for the procedure, blood group was established, and coagulation parameter assessment, blood morphology tests, chest X-ray, and abdominal ultrasound (ultrasound since 1985) were performed. Patients were fasting before the procedure. A transthoracic approach was routinely used, with the patient in the supine position. All biopsies were blind and performed using Menghini-type suction needles, initially with multiple-use devices and then using single-use ones starting 1997, i.e. Hepafix® (B. Braun Melsungen AG, Germany) sets. Needle diameters ranged from 1.4 to 1.8 mm. A 2% lidocaine solution was administered for local anesthesia. In some cases, intramuscular diazepam was administered as premedication. General anesthesia was used for biopsies of children under 14 years of age. All biopsies were performed on hospitalized patients who remained under observation for 1 day following the procedure. All samples were evaluated by a single pathologist. The Scheuer scoring system was used for the assessment of fibrosis and inflammatory activity. A chi-square test was used for statistical analysis; P < 0.05 was considered statistically significant. Calculations were performed using licensed Statistica software, (Stat Soft Inc. Tulsa, OK, US), version 8.0.

## 4. Results

We analyzed data from 1110 patients on whom 1412 percutaneous thick-needle blind liver biopsies were performed. Demographic characteristics of the study population are shown in Tabulation 1. The group included 699 male and 411 female patients; 888 biopsies (62.9%) were performed on males and 524 (37.1%) on females. The median patient age was 37 years (range, 2-72 years). Indications for liver biopsy are presented in Tabulation 2. Among 1412 liver biopsies performed, tissue samples were obtained from 1346 cases (95.3%), of which 1295 cases (91.7%) were fully representative as assessed by the pathologist. The obtained tissue samples did not allow for an accurate description of histological changes in 51 cases (3.6%) because of the small number of portal tracts. Tissue samples were not obtained from 66 (4.7%) biopsies. A total of 1153 (81.7%) biopsies were uncomplicated, while complications occurred in 259 biopsies (18.3%). No fatalities associated with biopsies were noted. Nine serious complications (0.64%), directly related to biopsy, were reported as follows: 5 cases of hemorrhage to the peritoneal cavity, 2 cases of hemothorax of the right pleural cavity, and 2 cases of biliary peritonitis. Laparotomy was required in 3 cases (2 incidents of biliary peritonitis and 1 massive hemorrhage to the peritoneal cavity). Drainage was needed for 1 hemothorax. In summary, a total of 4 invasive interventions were needed, which accounted for 0.28% of all biopsies performed and 1.5% of all complicated procedures. Clinical data from 9 patients with serious complications are summarized in Table 1.

**Table 1:** Serious Complications of Liver Biopsy

	**Management**	**Indication for LB a**	**Histology**	**Fibrosis**	**Age, y**	**Operator Experience, y**	**PLT a (×109/L)**	**PT a, %**	**Past Surgical Procedures**
**Male**									
Hemorrhage in the peritoneal cavity	laparotomy	abnormal liver tests	cirrhosis	yes	27	4	138	65	no
Hemorrhage in the peritoneal cavity	conservative	hepatitis B	nonspecific degenerative changes	no	30	2	115	82	no
Biliary peritonitis	laparotomy	hepatitis C	chronic viral hepatitis	no	31	1	217	94	no
Biliary peritonitis	laparotomy	abnormal liver tests	toxic lesions	yes	49	2	138	84	yes
Hemothorax	conservative	abnormal liver tests	sample not obtained	NA^a^	52	1	79	61	no
Hemorrhage in the peritoneal cavity	conservative	hepatitis C	chronic viral hepatitis	yes	54	5	150	83	yes
**Female**									
Hemothorax	drainage	hepatitis C	chronic viral hepatitis	yes	31	6	397	103	yes
Hemorrhage in the peritoneal cavity	conservative	abnormal liver tests	nonspecific degenerative changes	no	33	5	233	91	yes
Hemorrhage in the peritoneal cavity	conservative	hepatitis B	chronic viral hepatitis	no	55	4	116	87	no

^a^ Abbreviations: LB, liver biopsy; NA, not available; PLT, platelets; PT, prothrombin time

**Tabulation 1:** Characteristics of the Study Population

	**Value**
Total Patients, No.	1110
Total Liver Biopsies, No.	1412
Sex, No. (%)	
Male	699 (62.9)
Female	411 (37.1)
Caucasian, %	100
Age, y	
Range	2–72
First biopsy, mean	35.9
All biopsies, mean	36.2
< 18 y, No.	
Patients	105
Biopsies	130

**Tabulation 2:** Indications for Liver Biopsy

	**Patients, No. (n = 1110)**
Chronic hepatitis B	442
Chronic hepatitis C	302
Co-infection HBV/HCV	22
Liver cirrhosis (other than viral hepatitis)	34
Autoimmune hepatitis	22
Primary biliary cirrhosis	27
Abnormal liver tests	119
Alcoholic liver disease	43
NAFLD a	17
Toxic liver injury	18
Hyperbilirubinemia	42
Other	22

^a^ Abreviation: NAFLD;nonalcoholic fatty liver disease

Pain at the site of puncture and/or right shoulder was the most common complication and accounted for 216 (15.3%) cases; of these, administration of analgesics was indicated in 88 cases. Other reported complications included the following: 29 (2.05%) cases of vasovagal reaction (syncope, reflex hypotension, transient bradycardia), 16 (1.13%) cases of bile duct puncture (without resulting in bile leak and biliary peritonitis), 10 (0.71%) cases of leukocytosis after biopsy (in 4 cases with clinical signs of infection, with antibiotic administration), and 3 other uncommon incidents (2 episodes of anginal chest pain and 1 epileptic attack). Symptoms and signs of all complications occurred during postbiopsy observations, not later than 24 hours after the procedure. In 76 biopsies of children, general anesthesia was used. There were no complications associated with anesthesia or any other serious complications in these cases. Significantly more complications were reported in females (22.1%) than in males (16.1%) (P = 0.005). Pain (18.5% in women and 13.4% in men) and vasovagal reactions (2.3% and 1.9%, respectively) accounted for this difference. The most serious complications were twice as common in males as in females, whereas the proportion of all biopsies performed on men compared with women was 1.7:1. Age did not influence the risk of complications. Biopsies were performed by 8 different operators who were specialists in infectious diseases. Their experience, which was evaluated in years of clinical practice, ranged from 0 to 19 years. Less experience (< 2 years) was significantly associated with a higher biopsy failure rate (P = 0.002). Statistical significance of the relationship between individual operator efficiency and complication rate (P = 0.000) and that between individual operator efficiency and failure rate (P = 0.002) was observed. The relationship of complications and failure rates according to operator and his/her experience is presented in Table 2.

**Table 2:** Complications and Failure Rates According to Different Options

	**Failures, No. (%)**	**P value**	**Complications, No. (%)**	**P value**
Operator a		0.000		0.000
A (n = 244)	11 (4.5)		76 (31.1)	
B (n = 17)	4 (23.5)		2 (11.8)	
C (n = 59)	9 (15.3)		4 (6.8)	
D (n = 135)	10 (7.4)		35 (25.9)	
E (n = 55)	3 (5.5)		8 (14.5)	
F (n = 148)	8 (5.4)		27 (18.2)	
G (n = 128)	5 (3.9)		7 (5.5)	
H (n = 593)	5 (3.9)		95 (16.0)	
Operator Experience, y a		0.002		0.126
< 1 (n = 73)	5 (6.8)		9 (12.3)	
1–2 (n = 232)	21 (9.0)		36 (15.5)	
3–5 (n = 318)	17 (5.3)		54 (17.0)	
> 5 (n = 756)	23 (3.0)		155 (20.5)	
Previous Surgical Procedures on Abdominal Cavity and Thorax b		0.017		0.187
Surgical procedures (n = 393)	27 (6.9)		81 (20.6)	
Nonsurgical procedures (n = 1010)	39 (3.9)		177 (17.5)	
Staging				0.027
0–1 (n = 769)			131 (17.0)	
2–4 (n = 163)			41 (25.2)	

^a^ 33 biopsies were without data

^b^ No data in medical documentation in 9 cases

A total of 393 patients had a history of at least 1 surgical procedure involving the abdominal cavity or thorax (27.8% of all biopsies), which may have potentially changed the topography of organs in the abdominal cavity. The most common procedures were appendectomy (190 cases) and cholecystectomy (105 cases). A total of 19 patients were operated for peritonitis for various reasons, and another nine for abdominal injuries. Table 2 summarizes the significance of previous surgical procedures on the safety and effectiveness of liver biopsy. Among the unsuccessful biopsies, ten cases had a history of massive surgical intervention involving the abdominal cavity or organs adjacent to the liver, including five open cholecystectomies, two cases of purulent peritonitis as a complication of appendicitis, one splenectomy after abdominal injury, one kidney transplantation, and one large bowel resection. Liver fibrosis was also considered a factor influencing the safety and effectiveness of biopsies. Since a large number of biopsies were performed before the introduction of the scoring systems to the practice in the center, the presence or absence of fibrosis and the stage of fibrosis were assessed separately. The presence of fibrosis had no influence on the biopsy failure and complication rates. In 386 samples, the stage of fibrosis was established using the Scheuer scoring system and no fibrosis was described in 546 samples. Failure rate did not depend on the stage of fibrosis; however, the complication rate was significantly higher with a higher stage of fibrosis, as depicted in Table 2. In cases with early or complete cirrhosis, 25.3% of procedures were complicated, with 1 incident of hemorrhage in the peritoneal cavity.

## 5. Discussion

Knowledge related to the risk of complications in liver biopsy is based on several large analyses, which were performed mainly in the 1970s-1990s, cited by majority of papers concerning liver biopsies. Most studies were retrospective [10][13][14][15], similar to the present study, and some were based on multicenter databases [2][5][16]. Few studies reported detailed prospective observations [3][11][17]. The analyzed groups were heterogeneous in terms of indications, underlying conditions, methods, needles used, and guiding techniques. Established risk factors for serious complications include liver cirrhosis, malignancy, advanced age, impaired coagulation, and number of passes [3][18]. The time period of the present study was comparable to those of the other large analyses, which provided the possibility of including biopsies performed with older biopsy devices, similar to other papers. Biopsy indications were diverse during that period; however, in more recent years, the main indication was chronic viral hepatitis. The overall rate of complications in the analyzed group was low (18.3%) and the small number of serious complications (9-0.64%) observed was comparable to that of the other large cohorts (0.22-0.75%) [1][2][4][11][15][16]. No fatalities were reported, which may be attributable to the absence of the serious risk factors listed above in majority of patients and careful consideration of indications and contraindications for the procedure. Moreover, the needle used was the Menghini-type suction needle, which carries a smaller risk of bleeding compared with cutting needles such as the Tru-cut needle used widely in other cohorts [5][17]. The small number of serious complications did not allow for a detailed assessment of risk factors. More common reporting of pain and vasovagal reactions in women, similar to that described in other papers [11], might be partly because of underreporting of pain by men, or possibly as the result of a greater emotional reaction to the invasive procedure by women, observed by authors in clinical practice. Data concerning pain incidence are discordant and varying (range, 1.16-30%) in different reports [2][5][13][19][20]. Severity of pain may be accurately evaluated only in prospective studies, based on pain scoring systems [21][22]. The incidence of pain, particularly in mild cases without the need for analgesic administration, obtained from medical documentation in retrospective analyses, is probably often underreported. Few studies analyzed in detail the factors related to liver biopsy effectiveness; they were mainly concerned with the assessment of the effectiveness of image-guided procedures compared to blind biopsies [10][11]. In the data presented here, all the biopsies were blind; therefore, a comparison of the present analysis with other analyses is impossible. The relatively high failure rate in the present analysis (4.7%) may partly be attributed to the use of the suction needle. Cutting needles used in a large proportion of other studies permit the collection of larger tissue samples [23]. Other authors have reported failure rates of 1-2.2% [10][17][19][24], although Gilmore noted 5% of failures despite ultrasound guidance in majority of procedures [5]. Although preliminary and not a detailed observation, this study presents a unique finding of statistically significant higher failure rate of biopsies in patients with previous surgical procedures involving the thoracic or abdominal cavity. No other study has performed such analysis or reported this significance. One study discussed the variability of the anatomy and topography of the liver and adjacent organs found in computed tomography scans. The authors concluded that this variability may potentially affect the safety and success rate of blind liver biopsies [25]. Previous extensive surgical operations or massive inflammatory processes (i.e. peritonitis) may have a similar impact on abdominal organ topography, and this group of patients may certainly benefit from an image-guided procedure. Such a recommendation is also presented in the current guidelines of the American Association for the Study of Liver Disease for liver biopsies [6]. Another interesting observation was the impact of operator experience and skill on the safety and success rate of liver biopsy. In the present analysis, complication rates differed according to the skill of the operators, but not their experience, which suggests the important role of personal skill and precision as qualifications for performing the procedure compared to experience. The higher number of complications noted in procedures performed by experienced operators may also be due to the fact that they performed more technically difficult biopsies or biopsies on patients with risk factors.

Some authors reported similar observations indicating the role of clinical experience in the effectiveness of the procedure [5][14], whereas others in a French multicenter analysis did not find any such relationship [11]. Data concerning the influence of operator experience on complication rate are ambiguous. Cadranel and Froehlich reported a higher complication rate in procedures performed by physicians during training compared with experienced operators [11][16]. On the contrary, Perrault and McGill did not observe this association [3][17]. There are several limitations in the interpretation of our findings. Our study was conducted over a relatively long time period, during which the available equipment had changed, namely with respect to the availability of ultrasound and single-use sets. The retrospective analysis of data may involve underreporting of minor symptoms, especially mild pain. A quantitative assessment of biopsy effectiveness was impossible because the specimen length was not recorded.

In conclusion, blind percutaneous liver biopsy is a safe and effective invasive procedure, provided that the indications, contraindications, and risk factors for complications and failure are considered carefully. Moreover, it is important that biopsies are performed by skilled and experienced operators. Certain groups of patients may benefit from image-guided procedures. The small complication rate established in the present study may convince patients who are afraid of undergoing liver biopsy. Our results, confirming the safety of liver biopsy, are particularly significant for clinicians currently, when noninvasive fibrosis assessment methods (biochemical markers, elastography) are more widely available and used instead of histological evaluation.
